# Insight into Functional Membrane Proteins by Solution NMR: The Human Bcl-2 Protein—A Promising Cancer Drug Target

**DOI:** 10.3390/molecules26051467

**Published:** 2021-03-08

**Authors:** Ameeq Ul Mushtaq, Jörgen Ådén, Tobias Sparrman, Mattias Hedenström, Gerhard Gröbner

**Affiliations:** Department of Chemistry, Umeå University, SE-901 87 Umeå, Sweden; ameeq.ul.mushtaq@umu.se (A.U.M.); jorgen.aden@umu.se (J.Å.); tobias.sparrman@umu.se (T.S.); mattias.hedenstrom@umu.se (M.H.)

**Keywords:** Bcl-2 membrane protein, NMR, drug screening

## Abstract

Evasion from programmed cell death (apoptosis) is the main hallmark of cancer and a major cause of resistance to therapy. Many tumors simply ensure survival by over-expressing the cell-protecting (anti-apoptotic) Bcl-2 membrane protein involved in apoptotic regulation. However, the molecular mechanism by which Bcl-2 protein in its mitochondrial outer membrane location protects cells remains elusive due to the absence of structural insight; and current strategies to therapeutically interfere with these Bcl-2 sensitive cancers are limited. Here, we present an NMR-based approach to enable structural insight into Bcl-2 function; an approach also ideal as a fragment-based drug discovery platform for further identification and development of promising molecular Bcl-2 inhibitors. By using solution NMR spectroscopy on fully functional intact human Bcl-2 protein in a membrane-mimicking micellar environment, and constructs with specific functions remaining, we present a strategy for structure determination and specific drug screening of functional subunits of the Bcl-2 protein as targets. Using ^19^F NMR and a specific fragment library (Bionet) with fluorinated compounds we can successfully identify various binders and validate our strategy in the hunt for novel Bcl-2 selective cancer drug strategies to treat currently incurable Bcl-2 sensitive tumors.

## 1. Introduction

Virtually every life process proceeds via biological membranes and their proteins. Integral membrane proteins are estimated to be encoded for by approximately 30% of the human genome and fulfill a wide range of functions most prominently the cellular communication with its outer environment [1]. Therefore, not surprisingly, membrane proteins are important drug targets [2], like the well-known GPCR (G-protein coupled receptor) family with its most famous member rhodopsin involved in vision [3]. Those membrane proteins are crucial in sensing small ligands (such as hormones, neurotransmitters, and even drugs) outside the cell and communicate this information to the cell’s interior to trigger a biochemical response. However, beside all those polytopic integral membrane protein families, there exists also a distinct class called “tail anchored” membrane proteins [4].

Those proteins are located in various eukaryotic organelles, where they exert a wide range of functions ranging from vesicle transport to apoptosis. Those proteins have an N-terminal soluble domain and a single transmembrane domain in common, namely a hydrophobic helix at the C-terminus required for post-translational membrane anchoring. Those proteins possess a conformational plasticity allowing them to convert from a soluble state into a membrane-embedded state to exert their function. A famous example for this type of behavior is the members of the Bcl-2 protein family involved in regulation of the intrinsic (mitochondrial) pathway of apoptosis by controlling mitochondrial integrity [5]. Their opposing multi-spanning key members, such as the anti-apoptotic (cell protecting) Bcl-2 protein and its counterpart the apoptotic (cell killing) Bax protein, share quite similar homology of tail-anchored proteins despite opposite function. While Bax is soluble in the cytosol and translocates only upon apoptotic activation to the mitochondrion, to form pores releasing apoptotic factors like cytochrome c [6], anti-apoptotic Bcl-2 family members have the opposite behavior [7]. The Bcl-2 protein itself is found to reside at the MOM and sequesters membrane-associated Bax to prevent membrane perforation and cellular death [8]. However, the precise molecular nature of this key mechanism to ensure cell protection is not yet clear, despite significant progress on the general regulation process [8,9].

The Achilles heel to elucidate the precise mechanism of those membrane proteins of the Bcl-2 family is the lack of structural insight into their function, including their inherent built-in conformational flexibility and their ability to change interaction partners. In addition, there are further challenges for structural determination for those proteins, since their functioning and interplay occur mainly in a membrane-embedded active state [8,10,11]. While structures exist for Bax in its soluble cytosolic state and soluble N-terminal nano-disc anchored Bcl-xL [12,13], there are no known atomic detail structures for the intact, insoluble human Bcl-2 protein in its membrane-embedded state, but only for a soluble, chimeric, and truncated version with 166 aa. For this Bcl-2 version an NMR-derived structure is available [14], and also crystal structures [15]. This truncated version provided valuable insights into the function of Bcl-2, but does not function by itself in vivo and lacks any regulative capabilities. Simply, this truncated Bcl-2 (166 aa versus 239 of full-length protein) lacks key functional features like its long, intrinsically disordered regulative loop region (FLD; flexible loop domain) and it does not interact with its target membrane, the mitochondrial outer membrane (MOM) due to the absence of an hydrophobic C-terminal part which makes intact Bcl-2 protein insoluble but membrane-active in vivo. For a long time production of full-length human Bcl-2 protein was difficult and the molecular mechanism of its function was therefore elusive at the sub-molecular level.

To provide structural information of the intact human Bcl-2 membrane protein, a prime target in understanding cancer and treatment resistance, we have developed an NMR-based strategy as presented here. This strategy exploits our recent success in producing this protein in mg-amounts and refold it into lipid bilayers and membrane-mimicking micellar systems in a functional state [16,17]. The strategy is based on previous work on integral membrane proteins where one keeps the advantage of solution NMR by transferring membrane proteins into membrane-mimicking environments by solubilizing them into suitable detergent systems but retaining the basic functional features [18,19]. However, this procedure requires not only the successful production of functional protein, but also its transfer as a functional protein into micellar systems, whose small size still enables structural information obtained by solution NMR. In addition, we have also developed a platform consisting of Bcl-2 protein variants, detergent screening, and temperature optimization. This platform can be used now to extract structural information from intact human Bcl-2 protein in a membrane-mimicking environment and screen its functionality by NMR-based assays by binding natural BH3 domains of apoptotic proteins. In addition, solubilized functional Bcl-2 can be used as a drug target system to screen for molecular fragments and drugs from compound libraries by ligand-observed NMR high-throughput techniques to identify novel hits and lead structures. This strategy presented here will pave the way to acquire atomic-level information and shed fundamentally new light onto the molecular mechanism of native full-length human Bcl-2 protein, as well as open up the gateway for novel Bcl-2 selective cancer drug strategies.

## 2. Results and Discussions

Membrane proteins are important drug targets, which make screening of chemical libraries and studies of drug binding to these targets crucial for drug discovery and structure-based drug design approaches. Since solution NMR is not accessible to protein-membrane complexes due to the large size (>MDa) one can apply solid-state NMR. However, the resolution and sensitivity is quite limited compared to the solution NMR. Therefore, we use here intact Bcl-2 and variants retaining specific functional subunits in membrane-mimicking micellar environments which are accessible to solution NMR to obtain high-quality NMR spectra of Bcl-2 while retaining its function. Large membrane proteins with multiple domains and large loop regions also suffer from poor resolution due to peak overlap. Therefore, optimization of detergents, temperature, and protein sequence length are required for solution membrane protein NMR drug discovery approaches.

### 2.1. NMR Spectra of Bcl-2 Protein: Choice of Micellar System and Temperature

**Figure 1:**
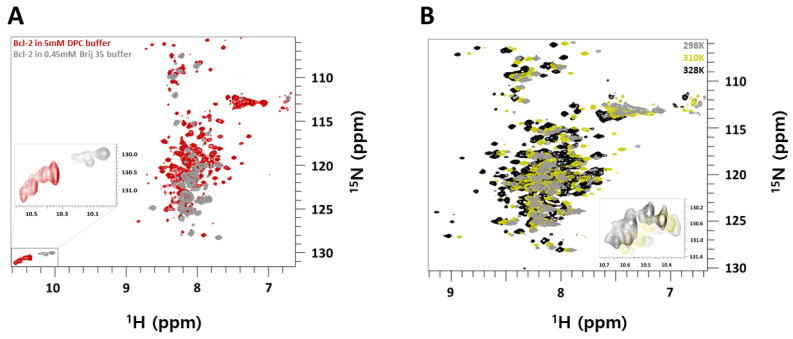
(**A**) Overlay of ^1^H-^15^N-TROSY-HSQC NMR spectra of 0.3 mM ^13^C/^15^N/^2^H-labeled Bcl-2 in 5 mM DPC micelles (red) in NMR buffer (20 mM NaPi, 20 mM NaCl, 2 mM TCEP at pH 6.0) and 0.35 mM ^15^N-labeled Bcl-2 in 0.45 mM Brij-35 micelles in NMR buffer at pH 7.4 (gray). All spectra were acquired at 298 K. (**B**) Overlay of ^1^H-^15^N-TROSY-HSQC NMR spectra of 0.36 mM ^15^N-labeled Bcl-2 at 298 K (gray), 310K (olive yellow), and 328 K (black) in 5 mM DPC micelles in NMR buffer. Zoomed box shows the tryptophan side-chain region of the spectra.

The spectral quality of NMR is critically dependent on the size and the tumbling rate of the studied system. Therefore, it is important to find a combination of membrane proteins and detergent which produce good sensitivity and spectral resolution while keeping the main functional feature of the protein intact. We tested a broad range of detergents as solubilization agents for the full length and truncated variants of the Bcl-2 protein, based on our previous work with cell-free expression systems. Out of this detergent screens which started with the detergent systems Brij 35 (starting point) the best outcome was DPC (Dodecylphosphocholine) with the result seen in Figure 1. Brij 35 itself is a non-ionic detergent with MW 1200, aggregation number(n) 40, CMC and micellar-size around < 0.1 mM and 48 kDa respectively. While DPC is a zwitterionic headgroup detergent with MW 351, aggregation number(n) 54, CMC and micellar-size around 1.5 mM and 19 kDa respectively [20,21]. As the starting point we used Brij-35 detergent, which turned out to be ideal for purification of intact human Bcl-2 protein by cell-free and standard E. coli overexpression systems [17,22] prior any further detergent exchange e.g., in DPC micelles. In those micellar environments, the Bcl-2 protein was fully functional as shown by a very high affinity constant (K_D_ around 35.8 nM) upon titration of its natural inhibiting partner, the soluble apoptotic Bax-protein [16]. This detergent system also enables the proper reconstitution of intact Bcl-2 protein back into its native lipid membrane environment.

However, as clearly seen here in Figure 1A, the corresponding ^1^H-^15^N-TROSY NMR spectrum of Bcl-2 in Brij-35 micelles at 298 K, reveals only a fraction of peaks well resolved (ca. 52 peaks) while the remaining ones are broadened or undetectable due to the slow tumbling rate of quite large Brij-35 micelles. Therefore, the spectral quality is not sufficient to identify most resonances of the Bcl-2 protein, which also makes it insufficient for any NMR-based structural work. Only the sharp resonances which belong to the extended (32–90) intrinsically disordered loop region FLD (as shown further down) can be clearly identified in the spectrum as separate peaks. By changing the detergent system to DPC which forms smaller micelles with faster tumbling rates a different picture is emerging. As seen in Figure 1A, the NMR spectra of the Bcl-2/DPC system reveal many more identifiable peaks (ca. 165 peaks) compared to Brij-35 under similar conditions. Clearly, the smaller size of the micellar systems with its increased tumbling rate provides significant advantages to investigate larger membrane proteins in a membrane-mimicking micellar environment by solution NMR methods. In the corresponding ^1^H-^15^N-TROSY NMR spectrum (Figure 1A, red) not only the peaks of the FLD domain are visible but also most resonances of the main folded domains composed of the N-terminal head (BH4 + BH3-1 domain). Deuteration of the protein did increase this number slightly compared to the non-deuterated Bcl-2 protein at 298 K, but not at higher temperatures, which improved both sensitivity (due to fast tumbling) and peak resolution of spectra for non-deuterated Bcl-2 with an optimum at 328 K. For non-deuterated Bcl-2 at the physiological temperature (310 K) and above at 328 K around 170 and 190 backbone signals were observed in the ^1^H-^15^N-TROSY spectrum respectively. Since full-length Bcl-2 protein consists of 25% of residues belonging to it intrinsically disordered FLD sequence, signals from this FLD region overlap with signals from the remaining protein causing severe peak overlapping in the 2D ^1^H-^15^N TROSY spectra. Combined with experiments at physiological temperatures it creates a bottleneck for structure-characterization due to the less sensitive ^13^C Cβ/Cα signal-intensities (for membrane-embedded BH1-3 domains) in 3D experiments. Therefore, increasing the temperature to 328 K (which increases tumbling rates for the temperature-stable Bcl-2 micelle system), proved advantageous since it provides a good signal to noise ratio of ^13^C signals from the side-chains of amino acids in the triple-resonance experiments.

Another important parameter to improve the spectral quality of Bcl-2 in DPC micelles is temperature. By increasing the temperature the overall tumbling rate becomes faster, providing better resolved NMR spectra. In addition, the increase of temperature also has a positive effect on the molecular dynamics of chemical moieties which reside on an intermediate NMR time scale at lower temperature, often connected with broadened or even non-detectable resonances. Moving those motional processes (e.g., local exchange or wobbling of molecular segments) into the fast region of the NMR time scale produces well resolved NMR spectra. As seen in Figure 1B, by increasing in temperature, the number of NMR peaks in the ^1^H-^15^N-TROSY spectrum also increases, with each peak representing one of the maximum 239 aa in the human Bcl-2 construct. Since rising temperature causes a faster tumbling rate of protein: micellar complexes, a comparison is shown in Figure 1B for temperatures 298 K, 310 K, and 328 K. Bcl-2 protein in 5 mM DPC buffer was stable at 328 K for nearly two weeks and upon cooling to 310 K or 298 K similar spectra were produced indicating that Bcl-2 protein in DPC micelles can maintain its stability above the physiological temperatures required for structure characterization with solution state NMR.

At higher or above physiological temperatures (>310 K), Bcl-2 or its truncated variants retain the folded structures in DPC micelles observed by the temperature dependent Far-UV Circular Dichroism (CD) measurements (Supplementary Figure S1A–E). Also, comparing the ^1^H-^15^N-TROSY spectra of Bcl-2 at 5 mM DPC (above CMC) conditions with the ones with below CMC at 0.25 mM DPC (see Supplementary Figure S2), indicate that the protein is still folded below the CMC of the detergent, thus generating very similar spectra. Refolded Bcl-2 or the truncated variants hold large amounts of DPC molecules. ^31^P NMR spectra of refolded Bcl-2 or the truncated variants in 5 mM DPC micelles in NMR buffer supports this observation when comparing spectra with and without DPC present (Supplementary Figure S3A,B). This strong lipid affinity toward the Bcl-2 structure is very important in the process of the MOM insertion. The embedded Bcl-2 state can be observed with NMR starting with the soluble fraction of Bcl-2 ∆TM. With ^1^H-^15^N-TROSY-HSQC spectra we observe the structural transition in Bcl-2 from a soluble to membrane-mimic embedded state, when the soluble fraction of Bcl-2 ΔTM was treated with different concentrations of DPC to mimic the membrane environments. At 0.5 mM DPC there were only specific local changes in the protein backbone and tryptophan side-chain ^15^Nε-^1^Hε cross-peaks (Supplementary Figure S4B). At 10 mM DPC, an increase in the concentration from below CMC to above CMC, significant changes were observed globally in the protein, and the tryptophan side-chain ^15^Nε-^1^Hε cross-peaks show a pattern similar to the refolded Bcl-2 ∆TM in 5 mM DPC buffer (Supplementary Figure S4A,B), indicating a structural-transition from a soluble to membrane-embedded state. We performed the functional assay of Bcl-2 and Bcl-2 truncated variants (Bcl-2 ∆TM, Bcl-2 ∆N(1–82), and Bcl-2 ∆N(1–82) ∆TM(208–239)) with conserved BH3-1 domain in membrane-mimicking environments using the peptide from the BH3 domain of Bax. Binding titrations were performed under similar buffer and experiment conditions to investigate the chemical shift perturbations (CSP’s) due to peptide binding. Binding titration results show that the BH3-1 domain is important for BH3 peptide binding. In addition a comparison between the binding of Bcl-2 ∆N(1–82) and Bcl-2 ∆N(1–82) ∆TM(208–239) shows that the TM domain also contributes to the binding affinity. (Supplementary Figure S5A–D). In Supplementary Figure S5E, CSP of backbone amide (^1^H, ^15^N and weighted ^1^H-^15^N) along the Bax peptide molar ratio is plotted for three peaks 1-3 (shown as boxes in Supplement Figure S5A,C) to highlight the differences upon binding titrations of full-length Bcl-2 and Bcl-2 ∆N(1–82). We observe similar patterns for the Bax peptide bindings in full-length Bcl-2 and Bcl-2 ∆N(1–82); however due to severe peak-overlaps in full-length Bcl-2 analysis becomes more difficult, while titrating the Bcl-2 ∆N(1–82) construct titration yields more information as there are no severe peak-overlaps. Plots of CSP along the Bax peptide molar ratio for the few backbone residues (peaks 1–3) show similar binding patterns with small differences in the affinities. However, the dynamic interplay or coordination between BH4 and BH3-1 TM domains via the FLD which has important significance for binding and inhibiting apoptotic full-length Bax can only be observed in full length Bcl-2.

### 2.2. The Effect of Bcl-2 Truncation on NMR Spectra

Since the full length 26kDa Bcl-2 protein is insoluble, a complete peak identification based solely on solution NMR spectra upon solubilization into micelles is still not straightforward. This is partially due to the micellar size but also that the Bcl-2 protein is composed of functional domains that generate partially overlapping NMR signals. Therefore, we have developed a “divide-and-conquer” strategy, where we produced constructs of Bcl-2 where specific domains and their functions were removed while the main protein core—its BH3-BH1 groove responsible for the sequestering of apoptotic Bcl-2 proteins—remained intact.

**Figure 2:**
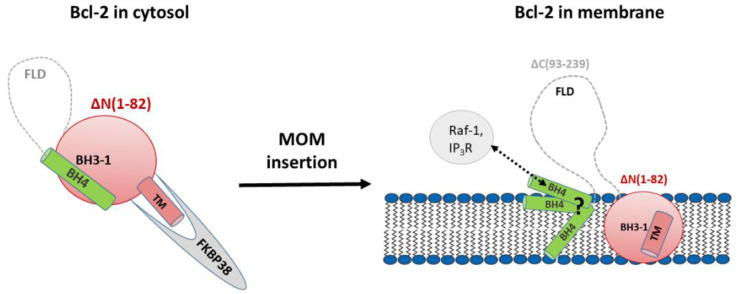
Our working concept: Schematic representation of the full length Bcl-2 sequence showing soluble Bcl-2 with the mitochondrial chaperone FKBP38 binding via the TM domain in the cytosol (left), which helps to transport Bcl-2 to the MOM by a still mysterious mechanism [26,27]. In the membrane, the Bcl-2 domain organization (right) based on the functions is shown as N-terminal BH4, FLD, and C-terminal BH3-1 TM domains. N-terminal domains are known to interact with IP_3_R and Raf-1 proteins [23,24,25], while C-terminal BH3-1 domains are known to interact with BH3-only proteins, interactions which are involved in apoptosis.

This strategy is based on our concept of Bcl-2 functioning based on the previous findings [23,24,25] as outlined below and schematically visualized in Figure 2.

Truncated Bcl-2 constructs with its extended FLD domain and hydrophobic TM-helix removed were shown to be soluble [14]. The Bcl-2 ∆TM construct with its hydrophobic TM-helix removed is partially soluble. This soluble escape (partial solubility) of Bcl-2 ∆TM can be explained based on the crystal structures of truncated soluble Bcl-2 variants where BH4 and BH3-1 domains are non-covalently packed while the FLD or truncated loop is extended to the outside of Bcl-2, and is also seen in other systems such as Bcl-xL [13]. Upon membrane insertion, the Bcl-2 domain organization based on the functions are shown as N-terminal BH4, FLD, and C-terminal BH3-1 and TM domains. N-terminal domains are known to interact with IP_3_R and Raf-1 proteins [23,24,25], while C-terminal BH3-1 domains are known to interact with BH3-only proteins, which are linked to apoptosis.

**Figure 3:**
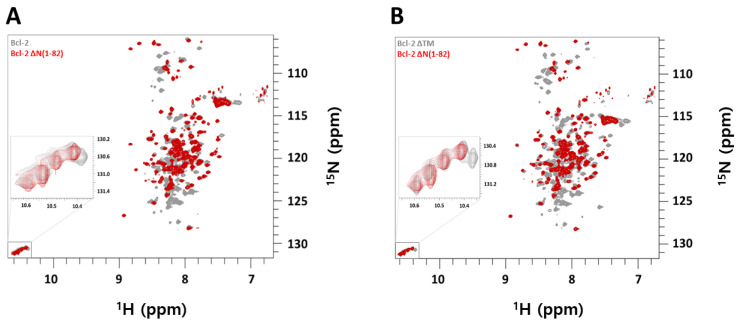
(**A**) Overlay of ^1^H-^15^N-TROSY-HSQC NMR spectra of 0.36 mM ^15^N-labeled Bcl-2 (gray), 0.4 mM ^15^N-labeled Bcl-2 ∆N(1–82) (red). (**B**) Overlay of ^1^H-^15^N-TROSY-HSQC NMR spectra of 0.7 mM ^15^N-labeled Bcl-2 ∆TM (gray) and 0.4 mM ^15^N-labeled Bcl-2 ∆N(1–82) (red). In all cases, conditions were 5 mM DPC micelles in NMR buffer at 310 K. The figure inserts (zoomed boxes) show the tryptophan side-chain region of the respective NMR spectrum.

Here we have now dissected Bcl-2 into constructs with either the TM domain missing (Bcl-2 ∆TM) or the BH4 + FLD region (Bcl-2 ∆N(1–82)). As seen in Figure 3A, Bcl-2 contains a long regulative flexible loop domain (FLD; aa 32–90) which shows overlapping peaks in the ^1^H-^15^N-TROSY spectrum with resonances from the protein’s main core body (BH3-1 and TM). By comparing the spectrum with the one obtained for Bcl-2 (Figure 1A, red) with the Bcl-2 ∆N(1–82) version, one can clearly identify that these flexible N-terminal residues 1–90 comprise the most and intense signals in the spectrum of the full length Bcl-2 variant (grey). Most of those resonances originate from the loop region and the others from the N-terminal BH4 region attached to it. Most other NMR signals belong to the main protein body (93–239 aa) and are weaker and often broadened due to large molecular weight of the Bcl-2/DPC micellar complex. Clearly, the truncation of N-terminal loop residues (1–82) or ∆N(1–82) results in less overlapping NMR spectra with similar peak patterns as the full Bcl-2 at 328 K. Therefore, truncation of the N-terminal loop residues (1–82) improves the spectral quality required for structure characterization of full Bcl-2 and probes structural changes upon interaction with other partners, ranging from BH3-domains of apoptotic proteins to promising drug targets. Since the TM domain remains, the functionality of the main protein fold is not compromised since this domain still contributes to the high affinity of the hydrophobic groove region [28].

Nevertheless also Bcl-2 without its TM domain is still functional as shown previously [28], albeit with less affinity toward BH3 domains compared to full-length Bcl-2 (our observations). As seen in Figure 3B (grey) the NMR spectrum of Bcl-2 ∆TM still shows an impact on the globular fold of the folded protein like the full-length one. Adding its spectrum together with the one obtained for Bcl-2 ∆N(1–82) (Figure 3B (red)) provides in summary an overlaid spectrum which is identical to the one obtained from the entire Bcl-2 protein itself (Figure 3A (grey). This clearly supports our “divide and conquer” strategy that dissecting Bcl-2 retains the structural features while keeping the specific functions intact. This is ideally not only for structural work of Bcl-2 in its membrane-mimicking micellar environment but also in the hunt for drug candidates specifically targeting one of those functional domains.

### 2.3. Domain Organization of Full-Length Bcl-2 under Membrane-Mimicking Conditions

**Figure 4:**
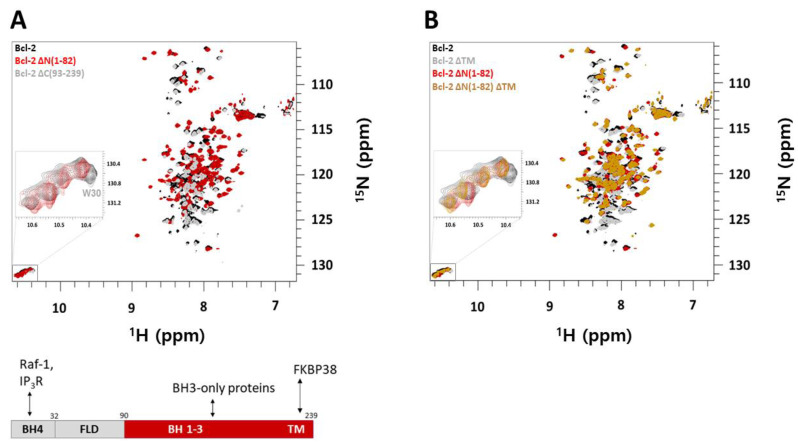
(**A**) Overlay of ^1^H-^15^N-TROSY-HSQC NMR spectra (top) of Bcl-2 constructs in 5 mM DPC micelles in NMR buffer at pH 6: (black) 0.36 mM ^15^N-labeled Bcl-2; (red) 0.4 mM ^15^N-labeled Bcl-2 ∆N(1–82); and (gray) 0.63 mM ^15^N-labeled Bcl-2 ∆C(93–239) at 310 K. Below is a schematic representation of the full length Bcl-2 sequence showing domain organization based on the function in its membrane. N-terminal BH4, FLD, C-terminal BH3-1, and N-terminal domains are known to interact with IP_3_R and Raf-1 proteins while C-terminal BH3-1 domains are known to interact with BH3-only proteins; interactions which are involved in apoptosis. The TM domain has been reported to be stabilized by the mitochondrial chaperone protein FKBP38 which actively translocate Bcl-2 from the cytosol to the MOM. (**B**) Overlay of ^1^H-^15^N-TROSY-HSQC spectra showing Bcl-2 (black), Bcl-2 ∆TM (gray), Bcl-2 ∆N1–82 (red), and Bcl-2 ∆N1-82 ∆TM (brown) in 5 mM DPC micelles in NMR buffer at 310 K. Overlay of the spectra shows that the structure is retained. Truncation of N-terminal and flexible loop residues (1–82) results in less overlapped NMR spectra. The Figure inserts (zoomed boxes) show the tryptophan side-chain region of the respective NMR spectrum.

Based on the functions of Bcl-2 in the membrane the Bcl-2 sequence is divided into an N-terminal BH4, FLD (aa 32–90) domain, and a C-terminal BH3-1 TM domain as shown in Figure 4 (bottom) and Supplementary Figure S6. N-terminal domains are known to interact with IP_3_R, Raf-1 [23,25] while C-terminal BH3-1 domains are known to interact with BH3-only proteins. Using Bcl-2 truncated variants together with NMR we concluded that N-terminal BH4, FLD, and the C-terminal BH3-1 TM domains are independent, since Bcl-2 ∆N(1–82) and Bcl-2 ∆C (93–239) together produce the full-length Bcl-2 ^1^H-^15^N-TROSY spectrum, shown in Figure 4 (top).

Since the full length Bcl-2 is insoluble while Bcl-2 ∆TM with the truncated transmembrane-helix is partially soluble, there is a soluble escape of Bcl-2 ∆TM where the truncated variant with its BH4 and BH3-1 domains are non-covalently fused with an extended flexible FLD or truncated loop. Further truncation of the N-terminal BH4 domain in Bcl-2 ∆TM also generated an insoluble Bcl-2 ∆N(1–82) ∆TM(208–239) construct (Supplementary Figure S6A,B). Therefore, using NMR we can target both functional sites of Bcl-2 for anti-cancer drug design, mimicking the membrane environments which is not possible with truncated soluble structures of Bcl-2 where the N-terminal BH4 helix is fused in the globular fold with the TM domain being absent. Those versions cannot probe independent functions of N-terminal BH4 and flexible loop domains (Supplementary Figure S6A).

This might be the solubility mechanism in cytosolic Bcl-2 homologues. When we further truncate the N-terminal BH4 domain in Bcl-2 ∆TM, the Bcl-2 ∆N(1–82) ∆TM construct was also insoluble (Supplementary Figure S6A,B), which shows that packing of the BH4 domain to the BH3-1 domain assembly is important for the proper formation of the globular fold of Bcl-2. Therefore, using intact Bcl-2 in membrane-like micellar environments is an ideal high-value cancer target where both the N- and C-terminal binding sites can be screened for promising drug candidates via high throughput screening methods based on solution NMR, where suitable substance libraries with the main principles are outlined under paragraph 2.4.

### 2.4. Drug Discovery: NMR Screening of Intact Bcl-2 Protein against Compound Libraries

**Figure 5:**
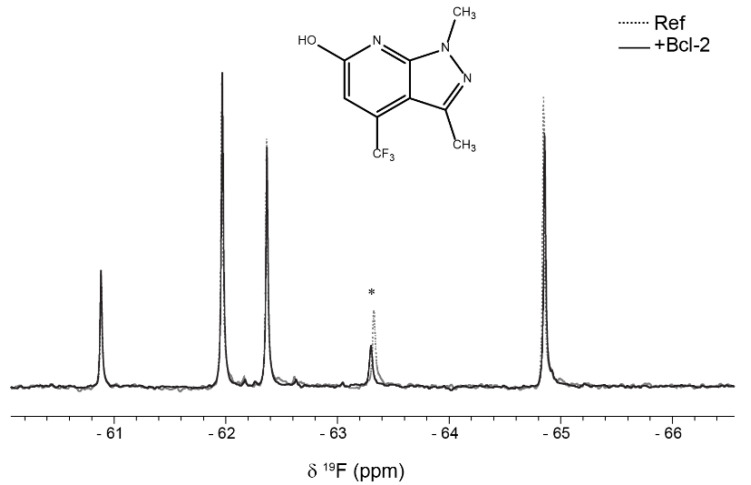
Zoomed region of ^19^F 100ms spin-echo spectra showing signals from five fragments (100 mM) in absence (dashed line) and presence (solid line) of 10 mM Bcl-2. Decreased signal intensity is observed for the peak marked with asterisk, identified as the fragment structure in the figure.

To provide new input into cancer therapy with Bcl-2 as the primary therapeutic target, fragment-based screening (FBS) using ^19^F NMR spectroscopy was performed. ^19^F NMR has several attractive properties compared to the more established ^1^H NMR used in FBS. Since the fluorine nucleus has a wide range of chemical shift values depending on its close molecular environment, it is normally not a problem to use mixtures of 10 or more fragments. Since those fragments are relatively simple and contain normally only one ^19^F nucleus, the individual fragments are easily identifiable in the corresponding spectra. Therefore, binding of a fragment to its target protein can simply be monitored as seen in Figure 5. Another advantage is that the NMR experiments can be performed in non-deuterated organic buffers. Moreover, binding response in T_2_-relaxation edited spectra are usually larger for ^19^F NMR because of the large chemical shift anisotropy of the ^19^F nucleus and a large line-width contribution from chemical exchange [29].

Binding to Bcl-2 was investigated using T_2_-relaxation edited experiments of fragment mixtures in absence and presence of Bcl-2 where a reduction in peak intensity was observed for fragments binding to the protein as a result of fast exchange between free and bound form. The samples recorded in absence of Bcl-2 still contained DPC micelles to detect fragments binding directly to the DPC micelle surface; fragments that otherwise would be detected as false positives.

**Figure 6:**
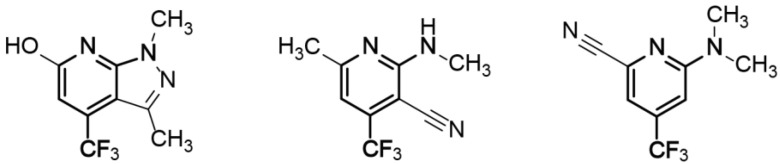
Three binding fragments exhibiting a common scaffold (highlighted in bold).

The commercially available Bionet fragment library, consisting of 428 fluorinated compounds was used in this study and screened in mixtures of 9–11 fragments per sample. Eight binding fragments were found, corresponding to a hit rate of approximately 2% (Supplementary Table S1). In addition, 11 fragments bound to the micelle surface as evident by no or very small ^19^F signal intensity in the reference samples. A common motif observed in 3 of the 8 binding fragments consists of a disubstituted pyridine ring with a CF_3_-group in the para position, see Figure 6. This scaffold could potentially be an interesting starting point for structure—activity relationship studies.

Hits will be further assessed for their potential by validating their binding and determining the affinities using biophysical methods (ITC, TSA) and by NMR on ^15^N labelled protein. As shown in Figure 3 and Figure 4, 2D ^1^H-^15^N NMR spectra can monitor binding of the molecule, its binding kinetics, and provide apparent binding affinities. Since each peak reflects a specific residue and its local environment we can assess if a binding event affects locally only the binding site (s. e.g., Figure 4) or if the entire protein undergoes structural rearrangements upon binding as seen in Figure 3. Here, the BH3 peptide has most effect on the residues directly involved in its binding (s. also inserts in Figure 3) but also the entire protein responds structurally (as seen by shifted resonances) to this binding event. The most affected residues presumably (ongoing assignment to confirm) belong to the Bcl-2′s binding groove (hotspot) and can be used to provide apparent affinities for a ligand upon titration; a strategy we will also exploit for new drug compounds to see if they interact to this key binding site of Bcl-2.

## 3. Materials and Methods

### 3.1. Preparation of Bcl-2 Protein and its Truncation Variants

Expression and purification of the Bcl-2 wild-type protein as well as the transmembrane deletion mutant Bcl-2ΔTM and N-terminal mutant Bcl-2ΔN(1–82) were accomplished by following the procedures as described previously [17], with the following exceptions: 1 L M9 media was prepared by adding 13 g KH_2_PO_4_, 10 g K_2_HPO_4_, 9 g Na_2_HPO_4_, 2.4 g K_2_SO_4_, 1.27 g MgCl_2_, 1 mL thiamine (30 mg/mL stock), 2 g ^12^C glucose or ^13^C glucose (Cambridge Isotope Laboratories, Inc., Tewksbury, MA, USA), and 2 g ^15^NH_4_Cl (Cambridge Isotope Laboratories, Inc., MA, USA). Prior to expression, the media was supplemented with trace elements (1 mL of 50 mM FeCl_3_, 20 mM CaCl_2_, 10 mM MnCl_2_, 10 mM ZnSO_4_, 2 mM CoCl_2_, 2 mM CuCl_2_, 2 mM NiCl_2_, 2 mM Na_2_MoO_4_ and 2 mM H_3_BO_3_ per liter medium), 100 μg/mL carbencillin and 34 μg/mL chloramphenicol, pH adjusted to 6.9 and sterile filtered. After binding to a His GraviTrap™ column (GE Healthcare, Little Chalfont, UK), the detergent Brij-35 was exchanged against 20 mM DPC (Dodecylphosphocholine, Glycon Biochemicals, GmbH, Luckenwalde, Germany) by applying ~50 column volumes of 20 mM Tris, 200 mM NaCl, 10% glycerol, 20 mM imidazole, 20 mM DPC, pH 7.8. Bcl-2 was eluted by using 10 mL of 20 mM Tris, 200 mM NaCl, 10% glycerol, 500 mM imidazole, 20 mM DPC, and pH 7.8. Successful elution of Bcl-2 protein was verified with SDS-PAGE. The protein was buffer exchanged against 20 mM Tris, 100 mM NaCl, 2 mM DTT, 20 mM DPC, and pH 7.8 using a PD-10 desalting column (GE Healthcare). To cleave the N-terminal His-tag, thrombin (GE Healthcare) was added in a 1:1250 (*w*/*w*) ratio and incubated over night at 4 °C. Final purification step of Bcl-2 was accomplished by gel filtration, using a Superdex 200 Increase 10/300 GL column (GE Healthcare), equilibrated with 5mM DPC in NMR buffer (20 mM NaP, 20 mM NaCl, 2 mM TCEP at pH 6.0).

To truncate the wild-type construct at position 207 (Bcl-2 ΔTM), the stop codon TAA was introduced by mutagenesis using the QuikChange approach (Stratagene). This was accomplished by forward primer 5′-GGACCTTCGATGCGTTAACTGTTTGATTTTTCG-3′ and reverse primer 5′-CGAAAAATCAAACAGTTAACGCATCGAAGGTCC-3′ (MWG Biotech, Ebersberg, Germany), respectively, using standard PCR routines. Successful insertion of the stop codon TAA was verified by DNA sequencing (MWG Biotech, Germany).

The gene coding for Bcl-2 ΔN(1–82) was purchased from GenScript^®^ (Leiden, the Netherlands) and sub-cloned into a pET-15b expression vector (Novagen). This deletes the first 82 N-terminal amino acids, making the N-terminus of the protein to start with residues MGP. The other Bcl-2 mutants were constructed using site-directed mutagenesis based on the Quikchange approach (Stratagene), conducted with standard PCR routines. For the Bcl-2 ΔN(1–82) ΔTM mutant, the stop codon TAA was introduced at position 207 by using the Bcl-2 ΔN(1–82) plasmid template, together with oligonucleotide forward primer 5′-GGACCTTCGATGCGTTAACTGTTTGATTTTTCG-3′ and reverse primer 5′-CGAAAAATCAAACAGTTAACGCATCGAAGGTCC-3′, respectively. This serves to create a Bcl-2 variant lacking both the C-terminal membrane binding domain and the first 82 N-terminal residues. Construction of the Bcl-2 ΔTM mutant was conducted using the same approach as above but using the full-length Bcl-2 protein as template, and introducing the TAA stop codon at position 208, thereby deleting the C-terminal residues and its membrane-binding domain. A similar site-directed mutagenesis approach was performed to create the Bcl-2 ΔC(93–239) mutant, here using the full-length Bcl-2 protein as template together with primers 5′-GTTCCGCCGGTGTAACATCTGACCCTGCGT-3′ and reverse primer 5′-ACGCAGGG-TCAGATGTTACACCGGCGGAAC-3′, respectively. This introduces the stop codon TAA at position 93, which deletes the remaining 146 C-terminal residues in the sequence. Proper insertion of the stop codon TAA was verified by DNA sequencing (Eurofins Genomics, Ebersberg, Germany). All oligonucleotide primers were purchased from MWG Biotech (Ebersberg, Germany). Important to note is that the full-length Bcl-2 and mutated constructs Bcl-2 ΔTM and Bcl-2 ΔC(93–239) all carry an additional three N-terminal residues, GSH, due to the design of the pET-15b expression vector. For concentration measurements, ε_280_ = 44,920 M^−1^ cm^−1^ was used for full-length Bcl-2, and for the mutants Bcl-2 ΔTM, ε_280_ = 37,930 M^−1^ cm^−1^, Bcl-2 ΔN(1-82) ε_280_ = 33,460 M^−1^ cm^−1^, Bcl-2 ΔN(1–82) ΔTM ε_280_ = 26,470, and Bcl-2 ΔC(93–239) ε_280_ = 11,460 M^−1^ cm^−1^ were used, respectively. Expression and purification of all constructs could be carried out in an identical manner.

#### 3.1.1. Far-UV Circular Dichroism (CD) Spectroscopy

CD spectra were recorded between 195 and 260 nm at 25 °C, 35 °C, 45 °C, 55 °C, 60 °C, and 70 °C using a Jasco J-720 Spectropolarimeter equipped with a Peltier temperature controller (Tokyo, Japan). The protein concentration in all cases was 5 μM in 5 mM DPC in NMR buffer. The spectra were recorded using a 0.1 cm quartz cuvette, a bandwidth of 2 nm with subtracted background, using 10 number of scans. Due to the high melting temperature of the Bcl-2 protein and mutated variants, any determination of melting temperature was not possible.

#### 3.1.2. Bax-BH3 Peptide Binding Assay

To check the functionality of the Bcl-2 protein and its variants, a Bax-BH3-derived peptide was titrated to those proteins in their detergent environment and binding was monitored by chemical shift perturbations using ^15^N TROSY-HSQC spectra (Supplementary Figure S5A–D). The 36-mer Bax-BH3 peptide from *Mus musculus* [17] (Ac-QPPQDASTKKLSECLRRIGDELDSNMELQRMIADVD-NH_2_) was ordered from GenScript (Piscataway, NJ, USA) and dissolved in 5 mM DPC containing NMR buffer, and added in a 1:1, 1:3, 1:6, and 1:12 molar ratio to 0.3 mM Bcl-2, 0.25 mM Bcl-2 ΔTM, 0.4 mM Bcl-2 ΔN(1–82), and 0.25 mM Bcl-2 ΔN(1–82) ΔTM samples respectively, followed by recording ^1^H-^15^N-TROSY-HSQC spectra with and without added peptide. All 2D ^1^H-^15^N TROSY experiments were performed with 8 number of scans, time-domain sizes of 256(^15^N) × 2048(^1^H) complex points and sweep widths of 11029.412 Hz and 2412.313 Hz along the ^1^H and ^15^N dimensions, respectively. All spectra were measured on 850 MHz magnet while for Bcl-2 ΔTM spectra were measured on 600 MHz magnet at 310 K.

### 3.2. NMR Spectroscopy on Bcl-2 Proteins

^1^H-^15^N TROSY (transverse relaxation optimized spectroscopy) experiments were performed using the following isotope labeled Bcl-2 proteins: 0.3 mM ^13^C/^15^N/^2^H-labeled full-length Bcl-2, 0.36 mM ^15^N-labeled full-length Bcl-2, 0.7 mM ^15^N-labeled Bcl-2 ∆TM, 0.4 mM ^15^N-labeled Bcl-2 ∆N(1–82) and 0.5 mM ^15^N-labeled Bcl-2 ∆N(1–82) ∆TM proteins in 5 mM DPC micelles in the NMR buffer 0.35 mM ^15^N-labeled Bcl-2 in 0.45 mM Brij-35 micelles in NMR buffer at pH 7.4, 40 μM ^15^N-labeled soluble Bcl-2 ∆TM in NMR buffer alone, and in 0.5 mM DPC and 10 mM DPC NMR buffers. All 2D ^1^H-^15^N TROSY experiments were performed with 16 or 32 number of scans, time-domain sizes of 256(^15^N) × 2048(^1^H) complex points and sweep widths of 11029.412 Hz and 2412.313 Hz along the ^1^H and ^15^N dimensions, respectively. For the 35 μM Bcl-2 and 25 μM ^15^N-labeled Bcl-2 ∆TM proteins below CMC, 0.25 mM DPC NMR buffer was used. All 2D ^1^H-^15^N TROSY experiments were acquired using 32 number of scans, time-domain sizes of 256(^15^N) × 2048(^1^H) complex points and sweep widths of 11029.412 Hz and 2583.979 Hz along the ^1^H and ^15^N dimensions, respectively. ^31^P NMR spectra were acquired under proton decoupling using a ^31^P pulse width of 12 µs duration and inter-scan delay of 5 s. NMR experiments were performed at 850 MHz if not stated otherwise (600 MHz) using Bruker Avance III spectrometers equipped with a triple-resonance TCI and BBO CryoProbes respectively. ^31^P NMR were acquired on 600 MHz Avance III spectrometer equipped with a BBO cryogenic probe. The pulse programs were obtained from the Bruker TopSpin 3.6.2library and data were processed and visualized using TopSpin 3.6 (Bruker biospin, Rheinstetten, Germany).

### 3.3. Fragment-Based Screening of Solubilized Bcl-2 Using ^19^F NMR

Samples were prepared so that a 10:100 molar ratio of Bcl-2 and fragments was achieved in the final samples, starting from 96-well plates containing 25 mM DMSO-d6 stock solutions of the 428 fragments. 1.6 µL of each fragment was added to 42 wells on a deep-well plate (plate A) filled with 378 µL 5 mM DPC in NMR buffer. This was done row-wise so that 9-11 fragments were added to each well (depending on the numbers of fragments in each row on the compound library plates). The solutions were mixed thoroughly then half of the volume was transferred to a new deep-well plate (plate B). Total of 2 µL of buffer (same as above) was added to all wells in plate A (these are the reference samples) and 2 µl of 0.72 mM Bcl-2 stock solution was added to the wells in plate B. Finally, samples were transferred to 3 mm SampleJet NMR tubes using a Bruker SamplePro pipetting robot.

All samples were analyzed at 298 K on a Bruker 600 MHz Avance III HD spectrometer equipped with a SampleJet (with cooling) and a 5 mm BBO cryo-probe. ^19^F-detected spin-echo experiments were performed on both the reference samples and samples with Bcl-2. A 4 ms 180 kHz smoothed chirp composite pulse was used instead of a square refocusing pulse to achieve uniform refocusing over the full sweep-width of 92 ppm [30]. Total of 512 scans were recorded for each spectrum using a total spin-echo time of 100 ms and a relaxation delay of 1.5 s. Spectra were analyzed using the FBS tool in Topspin 3.6 (Bruker biospin, Germany).

## 4. Conclusions

We here demonstrated both NMR-based structural approaches and anti-cancer drug discovery approaches to functional refolded Bcl-2 protein and its truncated variants in membrane-mimicking micellar environment. By exploiting intact Bcl-2 protein and truncation variants with specific functional domains solution NMR can be applied for structural studies. In addition, fully functional in vitro Bcl-2 protein (and its constructs for specific sub-functions) is a potentially powerful drug target for anti-cancer drug discovery; an application we demonstrated by screening against fluorinated chemical libraries using ^19^F and ^1^H-^15^N TROSY NMR to obtain the potential hits from diverse chemical libraries which can be further optimized with NMR SAR (Structure-activity relationships) to obtain promising anti-cancer drug candidates.

## Data Availability

The authors declare that all data supporting the findings of this study are available within the article, its Supplementary Information file and from the corresponding authors upon reasonable request.

## Supplementary Materials

The following are available online. Figure S1: Temperature stability of Bcl-2 and Bcl-2 truncation variants in DPC monitored by CD. Figure S2: 1H-15N-TROSY spectra of Bcl-2 protein above and below CMC of DPC. Figure S3: 31P NMR spectra of Bcl-2 protein in DPC micelles above and below CMC. Figure S4: NMR spectra of soluble and DPC titrated Bcl-2 ∆TM. Figure S5: In vitro binding assay on full Bcl-2, Bcl-2 ∆TM, Bcl-2 ∆N(1–82). and Bcl-2 ∆N(1–82) ∆TM constructs. Figure S6: Schematic diagram of Bcl-2 ∆TM showing truncation constructs. Table S1: Binding fragments identified by 19F NMR screening.

## Sample Availability

Plasmids of protein variants are available from the authors upon request.
