# Supplementary material for: Insight into Functional Membrane Proteins by Solution NMR: The Human Bcl-2 Protein—A Promising Cancer Drug Target
Source: Molecules. 2021 Mar 8;26(5):1467. doi: 10.3390/molecules26051467 (PMC7962812; doi:10.3390/molecules26051467)
Supplement: Supplementary file 1 [file molecules-26-01467-s001.pdf]

# Insight into Functional Membrane Proteins by Solution NMR: The Human Bcl-2 Protein – A Promising Cancer Drug Target

Ameeq Ul Mushtaq[1], Jörgen Ådén[1], Tobias Sparrman[1], Mattias Hedenström[1], Gerhard Gröbner[1]

[1] Department of Chemistry, Umeå University, SE-901 87 Umeå, Sweden

Correspondence to: gerhard.grobner@chem.umu.se

**Supplementary Information**

**Supplementary Figure 1**: Temperature stability of Bcl-2 and Bcl-2 truncated variants in DPC/NMR buffer monitored by CD.

**Supplementary Figure 2**: $^{1}H$-$^{15}N$-TROSY spectra of Bcl-2 protein in DPC/NMR buffer above and below CMC.

**Supplementary Figure 3:** $^{31}P$ NMR spectra of Bcl-2 protein in DPC/NMR buffer above and below CMC.

**Supplementary Figure 4**: NMR spectra of soluble and DPC titrated Bcl-2 ΔTM in NMR buffer

**Supplementary Figure 5**: (A-E): *In vitro* binding assays on full-length Bcl-2, Bcl-2 ΔTM, Bcl-2 ΔN (1-82) and Bcl-2 ΔN (1-82) ΔTM constructs**.**

**Supplementary Figure 6**: Schematic diagram of Bcl-2 ΔTM showing truncation constructs.

**Supplementary Table I**: Binding fragments identified by $^{19}F$ NMR screening.

***Supplementary Figure 1. Temperature stability of Bcl-2 and Bcl-2 truncated variants in DPC monitored by CD.***

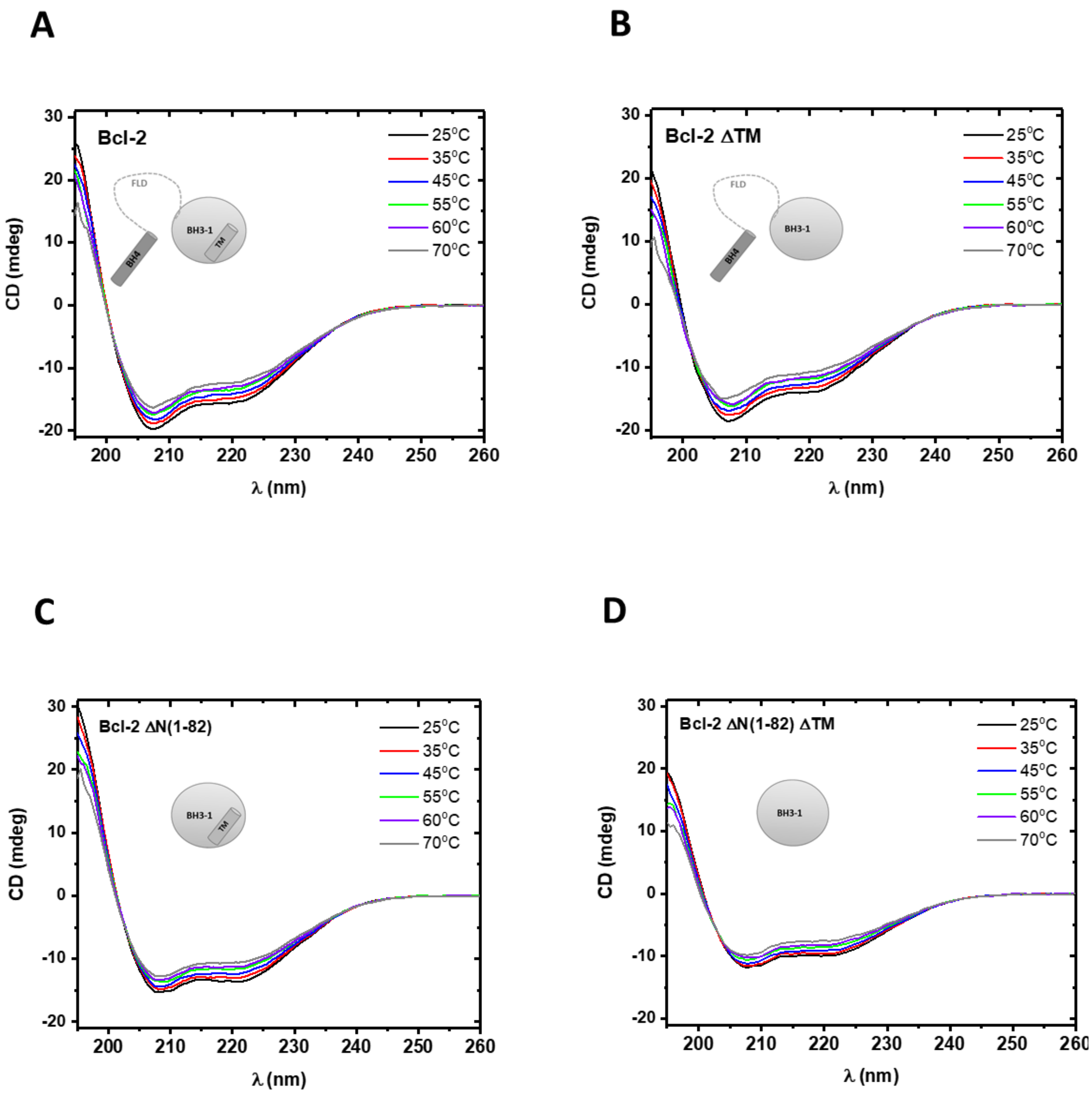

**E**

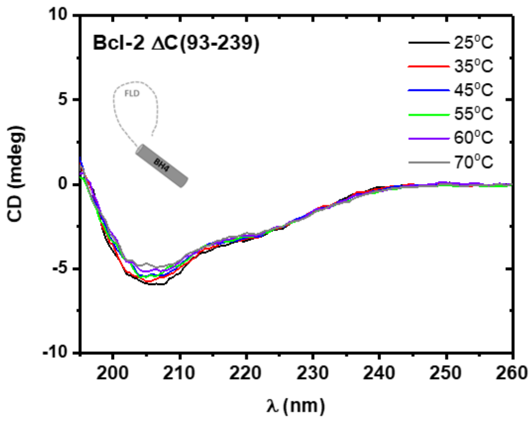


**Supplementary Figure 1**. **A-E)** Far-UV CD spectra of full-length Bcl-2 protein compared to truncated variants in 5mM DPC in NMR buffer (20 mM NaPi, 20 mM NaCl, 2 mM TCEP, pH 6.0), recorded at increasing temperatures between 25°C and 70°C. **A)** CD spectra of 5 µM full-length Bcl-2, **B)** 5 µM Bcl-2 ΔTM, **C)** 5 µM Bcl-2 ΔN(1-82), **D)** 5 µM Bcl-2 ΔN(1-82) ΔTM, and **E)** 5 µM Bcl-2 ΔC(93-239). All shown spectra are averages of 10 individual scans with subtracted buffer backgrounds.

***Supplementary Figure 2. NMR spectra dependence on CMC of DPC: spectra above and below CMC of DPC.***

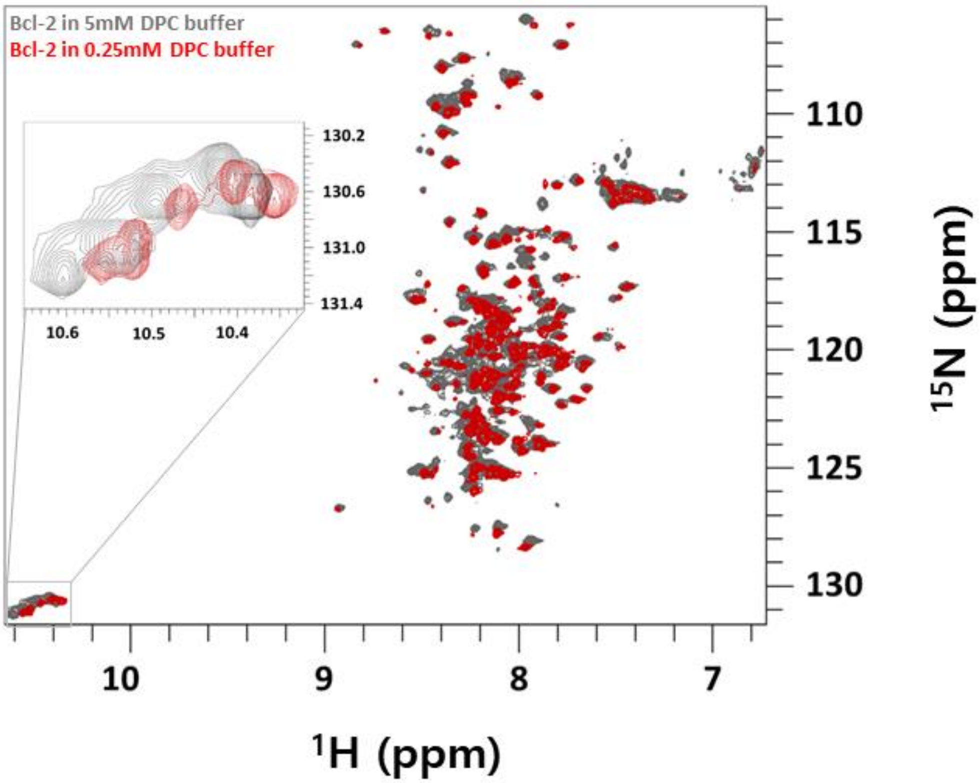


**Supplementary Figure 2**. Overlay of $^{1}$H-$^{15}$N-TROSY-HSQC NMR spectra of 0.36mM $^{15}$N-labeled Bcl-2 in 5 mM DPC micelles in NMR buffer (gray) and 35 µM $^{15}$N-labeled Bcl-2 in 0.25 mM DPC in NMR buffer (red); all at 310 K.

*Supplementary Figure 3. $^{31}P$ NMR spectra of Bcl-2 protein in DPC micelles above and below CMC.*

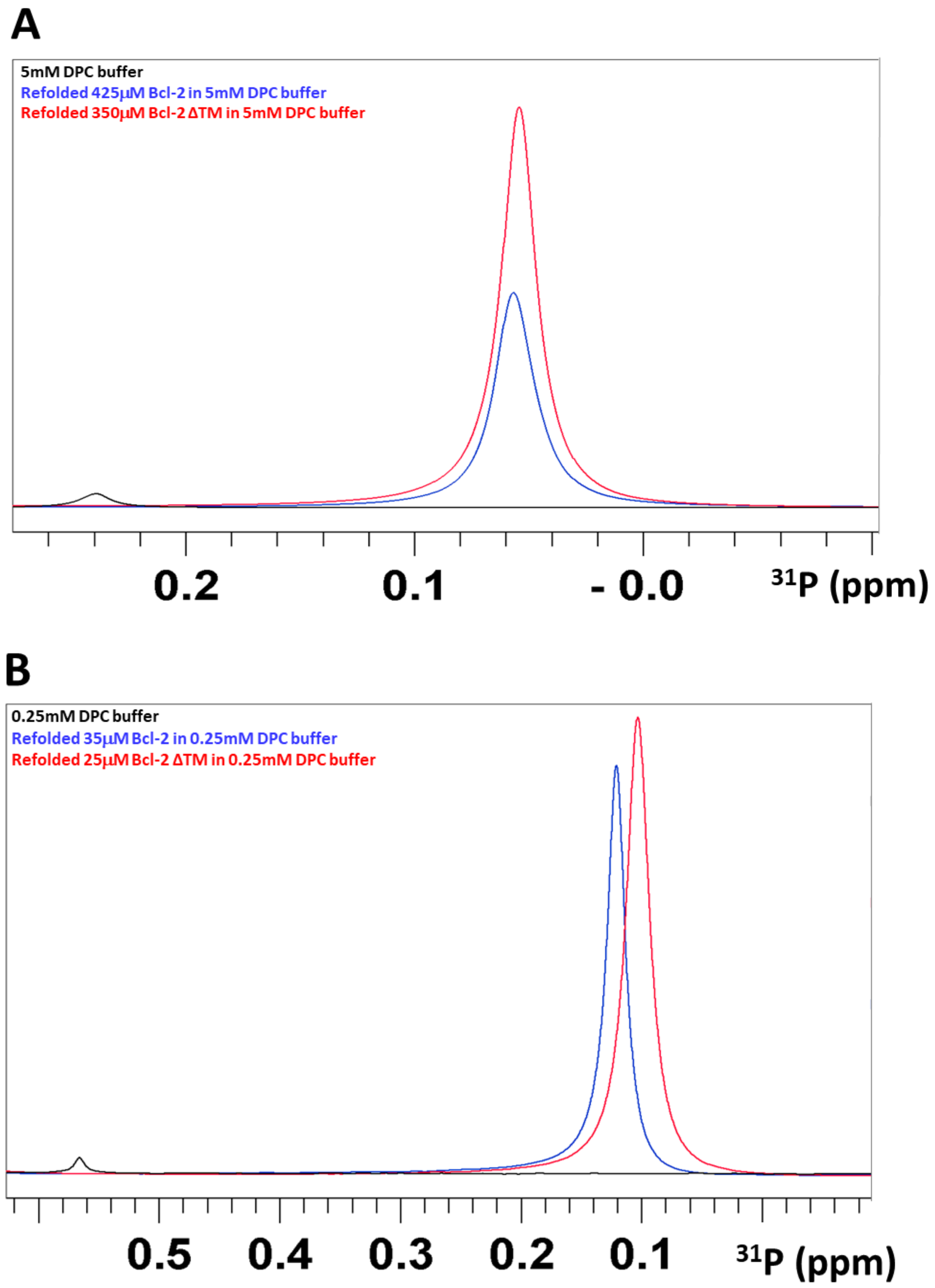

**Supplementary Figure 3**. **(A)** $^{31}P$ NMR spectra of reference 5 mM DPC micelles in NMR buffer(black), 425μM Bcl-2 and 350 μM Bcl-2 ΔTM in 5 mM DPC micelles in NMR buffer (in blue and red, respectively) at 298 K.

**(B)** $^{31}P$ NMR spectra of reference 0.25 mM DPC in NMR at pH 6.0 (black), 35μM Bcl-2 and 25 μM Bcl-2 ΔTM in 0.25 mM DPC in NMR buffer (in blue and red, respectively). NMR spectra were acquired under proton decoupling using a $^{31}P$ pulse width of 12 μs duration and inter-scan delay of 5 s.

***Supplementary Figure 4. Bcl-2 ΔTM soluble and DPC titrated (membrane-mimic environment) NMR spectra.***

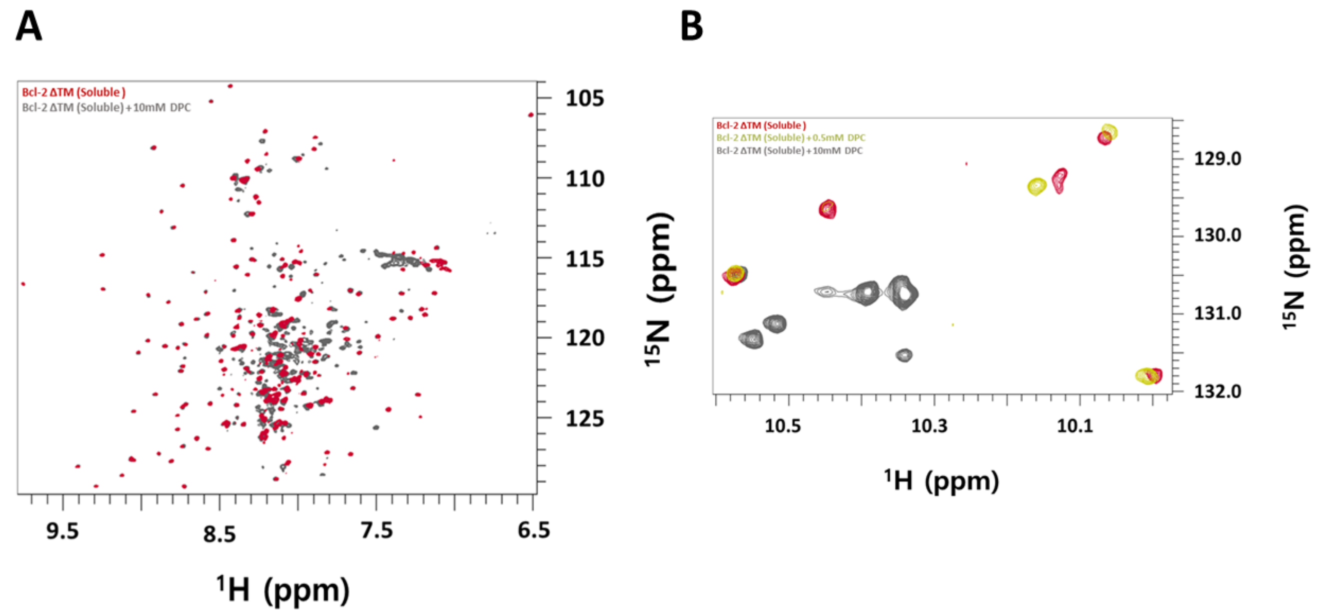


**Supplementary Figure 4. (A)** Overlay of $^{1}$H-$^{15}$N-TROSY-HSQC NMR spectra of 40 μM $^{15}$N-labeled Bcl-2 ΔTM in 20 mM NaPi, 20 mM NaCl, 2 mM TCEP at pH 6.0 (red), 40 μM $^{15}$N-labeled Bcl-2 ΔTM in 10 mM DPC micelles in NMR buffer(gray). **(B)** Overlay of $^{1}$H-$^{15}$N TROSY-HSQC spectra showing side-chain tryptophan $^{15}N\varepsilon^{1}H$ of 40 μM $^{15}$N-labeled Bcl-2 ΔTM in NMR buffer (red), 40 μM $^{15}$N-labeled Bcl-2 ΔTM in 0.5 mM DPC in NMR buffer (olive yellow), 40 μM $^{15}$N-labeled Bcl-2 ΔTM in 10 mM DPC micelles in NMR buffer (gray). All spectra acquired at 298 K.

***Supplementary Figure 5 (A-E). In vitro binding assay on full-length Bcl-2, Bcl-2 ΔTM, Bcl-2 ΔN (1-82) and Bcl-2 ΔN (1-82) ΔTM constructs.***

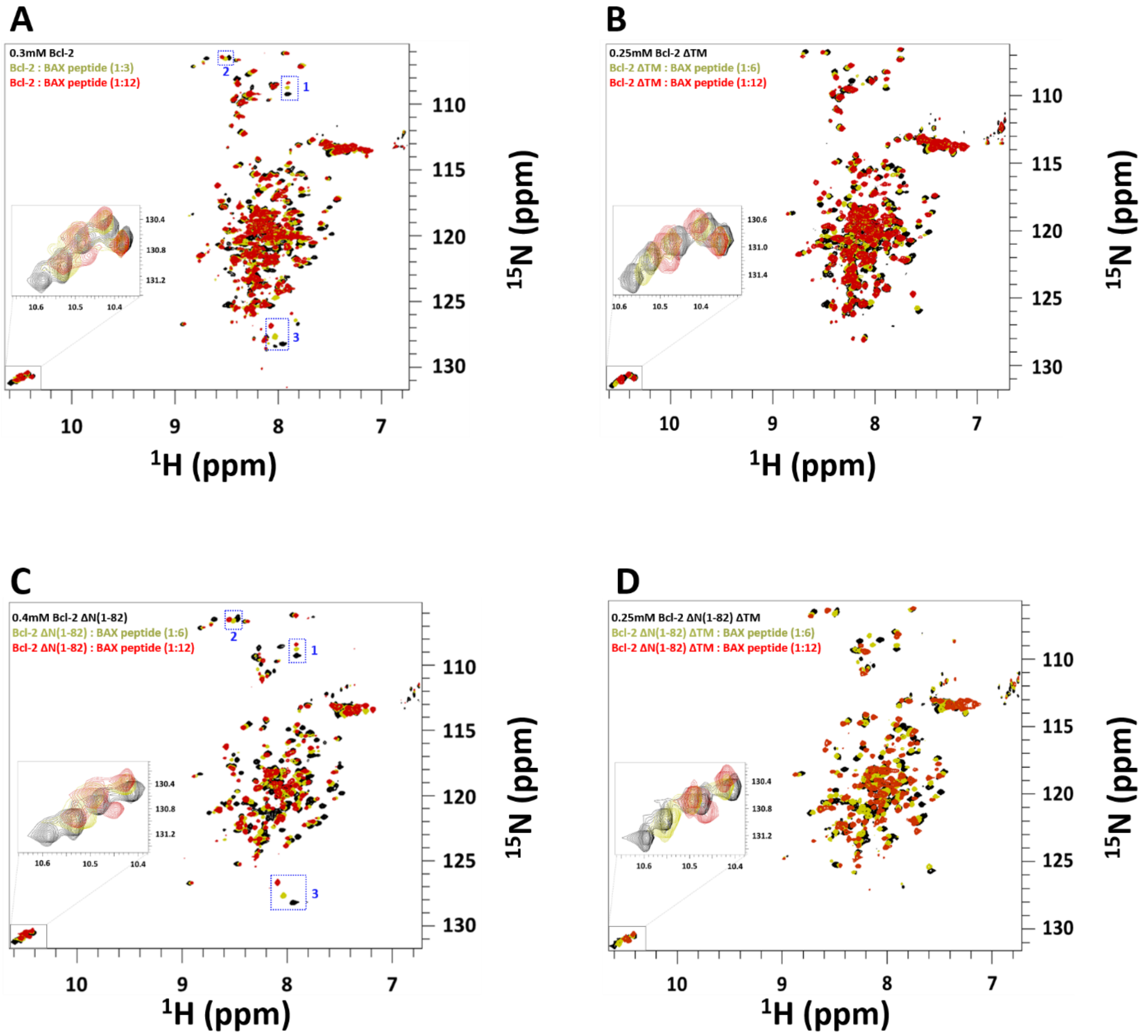


**Supplementary Figure 5 (A-D)**. **(A)** Overlay of $^{1}$H-$^{15}$N-TROSY-HSQC NMR spectra showing chemical shift perturbations (CSP's) observed in $^{15}$N-labeled Bcl-2 upon titration with the Bax-BH3 peptide at 310K. In black is the spectrum of 0.3 mM Bcl-2 in 5 mM DPC micelles in NMR buffer. Coloured in olive, yellow and red are the spectra of Bcl-2 with Bax-BH3 peptide added at 1:3 and 1:12 stoichiometry. **(B)** Overlay of $^{1}$H-$^{15}$N-TROSY-HSQC NMR spectra showing chemical shift perturbations (CSP's) observed in the $^{15}$N-labeled Bcl-2 ΔTM when titrated with Bax-BH3 peptide at 310 K. In black is the spectrum of 0.25 mM Bcl-2 ΔTM in 5 mM DPC micelles

in NMR buffer. In olive yellow and red are the spectra of Bcl-2 with Bax-BH3 peptide added at 1:6 and 1:12 stoichiometry. **(C)** Overlay of $^{1}$H-$^{15}$N-TROSY-HSQC NMR spectra showing chemical shift perturbations (CSP's) observed in the $^{15}$N-labeled Bcl-2 ΔN(1-82) when titrated with Bax-BH3 peptide at 310K. In black is the spectrum of 0.4 mM Bcl-2 ΔN(1-82) in 5 mM DPC micelles in NMR buffer. In olive yellow and red are the spectra of Bcl-2 with Bax-BH3 peptide added at 1:6 and 1:12 stoichiometry. **(D)** Overlay of $^{1}$H-$^{15}$N-TROSY-HSQC NMR spectra showing chemical shift perturbations (CSP's) observed in the $^{15}$N-labeled Bcl-2 ΔN(1-82) ΔTM when titrated with Bax-BH3 peptide at 310 K. In black is the spectrum of 0.25 mM Bcl-2 ΔN(1-82) ΔTM in 5 mM DPC micelles in NMR buffer. In olive yellow and red are the spectra of Bcl-2 with Bax-BH3 peptide added at 1:6 and 1:12 stoichiometry. The Figure inserts (zoomed boxes) show the tryptophan side-chain region of the respective NMR spectrum.

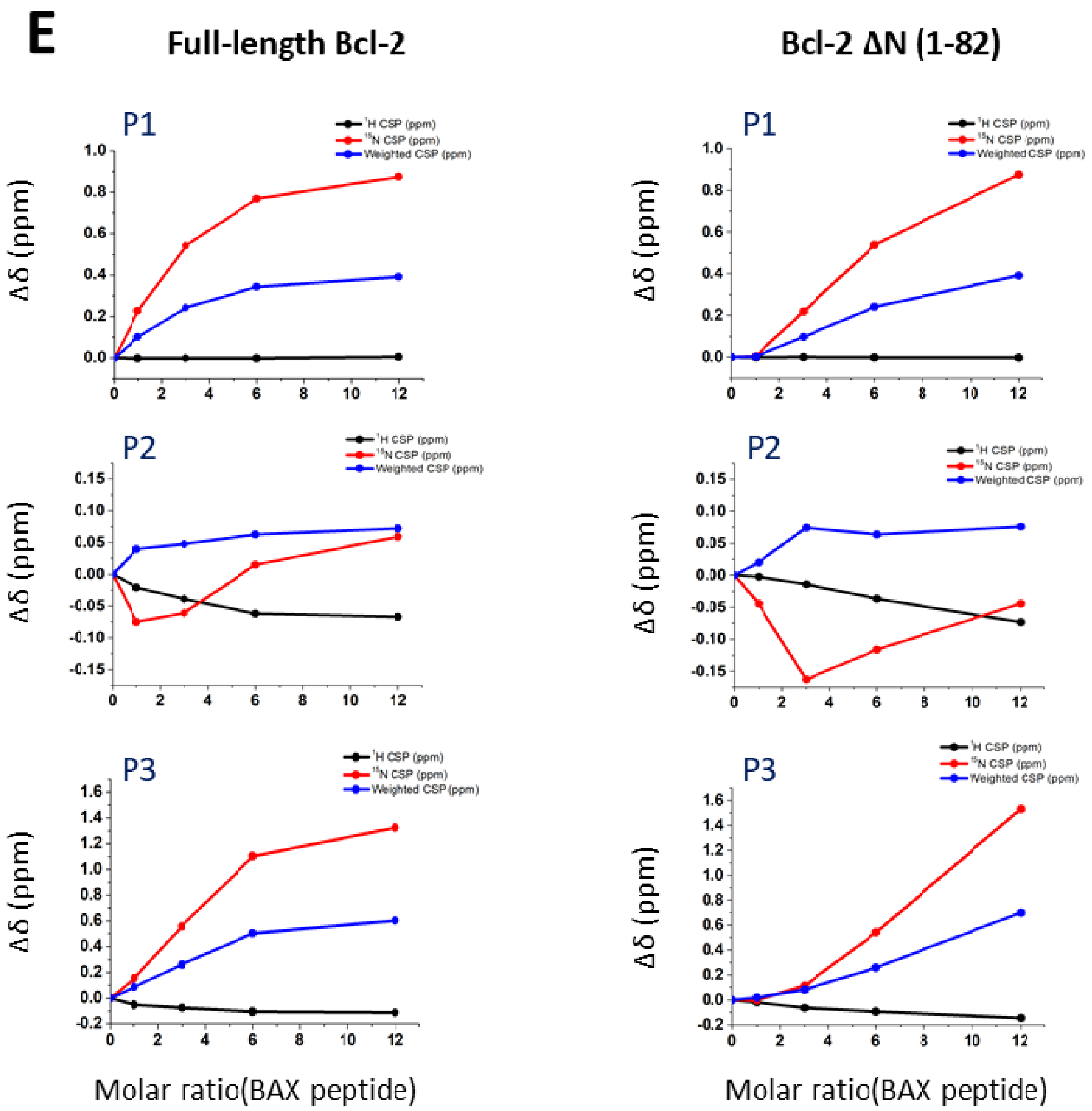


**Supplementary Figure 5E**. Plots showing the Chemical Shift Perturbation's (CSP's) of the $^{1}$H-N protons (δH), $^{15}$N amide nitrogen's(δN) and weighted $^{1}$H-$^{15}$N chemical-shift differences ($\Delta\delta_{1H\text{-}15N}$ (ppm) = $[\ (\delta H)^2 + 0.2 \times (\delta N)^2]^{1/2}$ ) of the backbone amide peaks (P1-3 highlighted as dashed boxes in Supplementary Figures 5A and 5C) of the Bcl-2 and Bcl-2 ΔN(1-82) upon titration with BH3 Bax peptide at Bax peptide/protein molar ratios of 0, 1, 3, 6 and 12, respectively.

***Supplementary Figure 6. Schematic diagram of Bcl-2 ΔTM showing truncation constructs***

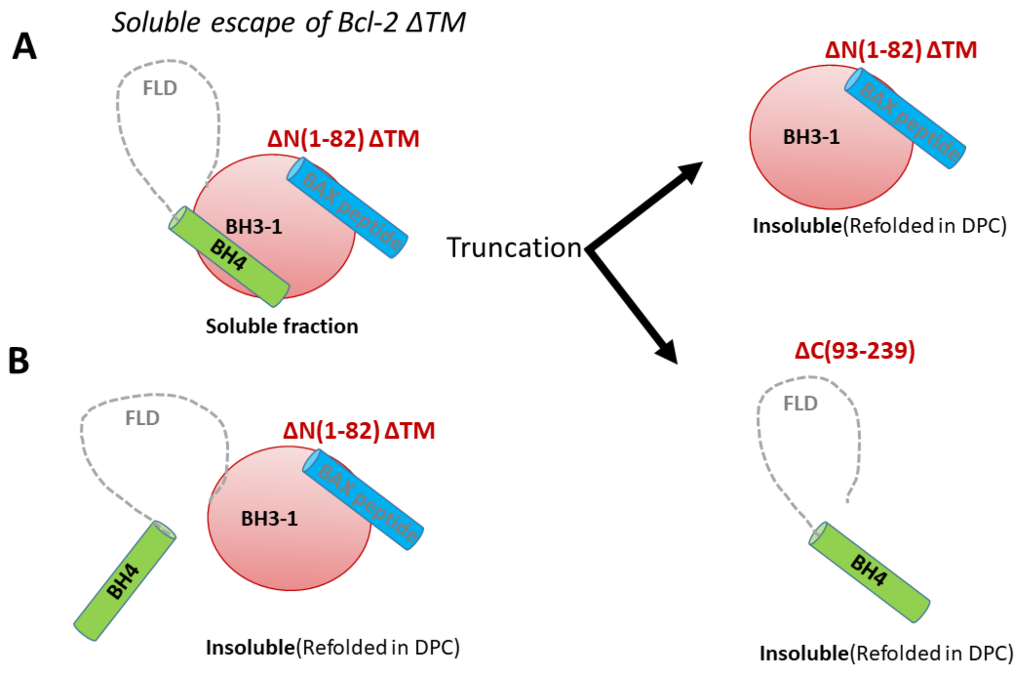


**Supplementary Figure 6**. **(A)** Schematic diagram showing the truncation of **(A)** soluble and **(B)** insoluble Bcl-2 ΔTM, soluble Bcl-2 ΔTM fraction showing BH4 and BH3-1 domains packed together to form a soluble globular fold.

**Supplementary Table 1. Binding fragments found in the $^{19}$F screening**

| Hit | Structure | $T_2$ response (%)[a] |
|---|---|---|
| 1 | 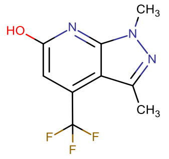 | 45 |
| 2 | 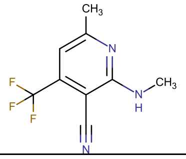 | 55 |
| 3 | 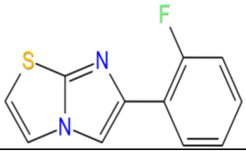 | 30 |
| 4 | 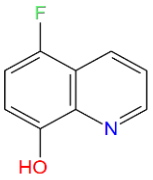 | 30 |
| 5 | 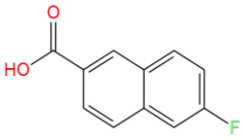 | 50 |
| 6 | 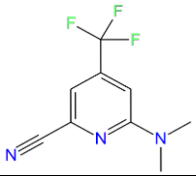 | 30 |
| 7 | 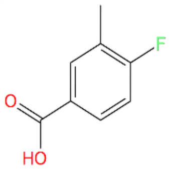 | 30 |
| 8 | 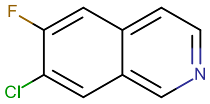 | 25 |

[a]The observed signal reduction in the spin-echo experiment in samples with Bcl-2 compared to reference samples.
